# Causal Relationship Between the Abuse of Cholesterol‐Lowering Medication, Blood Pressure Medication, Insulin, and Exogenous Hormones and Cerebral Infarction

**DOI:** 10.1002/brb3.70186

**Published:** 2024-12-22

**Authors:** Jing Liu, Dongsheng Lv, Shiduo Li, Zhansen An, Zefeng He, Yingzi Liu

**Affiliations:** ^1^ Department of Neurosurgery The Fourth Hospital of Hebei Medical University Shijiazhuang China

**Keywords:** blood pressure medication, causal association, cerebral infarction, insulin, Mendelian randomization

## Abstract

**Background:**

Cholesterol‐lowering medications, blood pressure medication, insulin, and exogenous hormones (including hormone replacement therapy, oral contraceptives, and minipills) are commonly utilized in clinical practice. Recent studies indicate that the use of these medications may significantly influence the occurrence and progression of cerebral infarction. This study aims to investigate the relationship between these medications and cerebral infarction using Mendelian randomization (MR) analysis, with the goal of offering valuable insights for the clinical management of cerebral infarction.

**Methods:**

To explore the correlation between cholesterol‐lowering medication, blood pressure medication, insulin, exogenous hormones, and cerebral infarction, relevant single nucleotide polymorphisms (SNPs) were extracted from the genome‐wide association study (GWAS) database. Methods for univariate MR analysis include inverse variance weighting (IVW), the weighted median method, and the MR‐Egger method, with IVW being the predominant approach. Subsequently, multivariable Mendelian randomization (MVMR) was conducted on the positive results obtained from the IVW analysis to verify the independent effect of each positive exposure, with IVW still predominating. The causal relationship between this class of drugs and cerebral infarction was evaluated using odds ratio (OR) and 95% confidence interval (CI). MR‐PRESSO was used to test for pleiotropy. The robustness of the findings was assessed through leave‐one‐out analysis, Cochran's *Q* test, and funnel plot.

**Results:**

Univariate MR results indicated that the use of blood pressure medication, insulin, and cholesterol‐lowering medication was significantly associated with the occurrence of cerebral infarction (all *p* < 0.05). However, due to the stringent inclusion criteria for SNPs, the number of available SNPs is insufficient to elucidate the association between exogenous hormone drugs, contraceptives, and cerebral infarction. Furthermore, the MVMR analysis, which builds upon univariate MR, only identified significant causal associations between blood pressure medication, insulin, and cerebral infarction (*p* < 0.05). The association between cholesterol‐lowering medication and cerebral infarction was confounded by other positive exposures and did not demonstrate a significant causal relationship when only its independent effects were considered. After integrating the findings from both univariate and MVMR and controlling for confounding variables to the greatest extent possible, the available evidence supports only a potential causal relationship between blood pressure medication, insulin, and cerebral infarction.

**Conclusion:**

This study suggests that the misuse of blood pressure medications and insulin is a risk factor for the occurrence and progression of cerebral infarction.

## Graphical abstract

Using Mendelian randomization analysis, we found that the abuse of blood pressure medication and insulin were potential risk factors for cerebral infarction. Excessive use of blood pressure medication and insulin can induce and aggravate cerebral infarction.

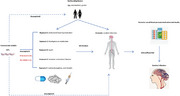

## 1 Introduction

Cerebral infarction refers to the necrosis of brain tissue resulting from inadequate blood supply to the brain or stenosis of cerebral vessels. It is the leading cause of disability worldwide (Feske 2021; Paul and Candelario‐Jalil 2021; Walter 2022). Patients with cerebral infarction frequently exhibit symptoms such as hemiplegia, altered consciousness, and speech disorders. These patients have a high recurrence rate and are challenging to treat clinically, presenting a significant public health challenge (Bhatia et al. 2023; Walter 2022). Although significant efforts have been made in clinical practice, there remains a shortage of effective medications for the treatment of cerebral infarction. Consequently, early prevention and control of risk factors are crucial. Cholesterol‐lowering medication, blood pressure medication, insulin, and exogenous hormones play vital roles in the management of cardiovascular diseases as well as in the regulation of endocrine and metabolic disorders. Some studies have indicated that the use of blood pressure medications, such as nimodipine (Rass et al. 2023), and cholesterol‐lowering medications, such as statins (Lee et al. 2020), may reduce the risk of cerebral infarction. However, in clinical practice, we have also observed that inappropriate or excessive use of blood pressure medications can trigger or exacerbate cerebral infarction. It is important to note that most of these studies are observational in nature, and the presence of confounding variables and biases may hinder the establishment of causality. Additionally, the relationship between insulin, exogenous hormones, and the risk of cerebral infarction remains unclear. Therefore, elucidating the correlation between cerebral infarction and these commonly used medications is essential for disease prevention.

Mendelian randomization (MR) has been extensively utilized to ascertain the causal relationship between different exposures and outcomes. In contrast to the conventional randomized controlled trials (RCTs), the MR approach allocates subjects randomly to either the treatment or control group based on its inherent principles (O'Donnell and Sabatine 2018). The addition of genetic tools can largely reduce the influence of confounding factors (Emdin, Khera, and Kathiresan 2017). When the conditions for RCTs are inadequate, reliable causal associations between exposure and outcomes can be established through MR analysis (Zuccolo and Holmes 2017). MR relies on the principle of random assortment of genetic variants during meiosis, ensuring that these variants are randomly distributed within the population. Mendelian inheritance models account for the genetic variants associated with exposure factors, and genetic variants are randomly paired during zygote formation, similar to an RCT process. Consequently, the causal relationship between exposure and outcome analyzed by MR is not influenced by gender, age, environmental factors, or other confounding variables (Burgess, Butterworth, and Thompson 2013), thereby enhancing the accuracy of the causal inference. Furthermore, genes are determined at birth and remain unaffected by disease progression, which minimizes the potential for reverse causality (Cai et al. 2022). During human gametogenesis, alleles of single nucleotide polymorphisms (SNPs) are randomly distributed in sperm. SNPs are commonly utilized as instrumental variables (IVs) to investigate the associations between traits in MR analysis. Therefore, by conducting a dual‐sample MR analysis, it can aid in determining the association between cholesterol‐lowering medication, blood pressure medication, insulin, exogenous hormones (such as hormone replacement therapy, oral contraceptive pill, or minipill), and the risk of cerebral infarction.

## 2 Materials and Methods

### 2.1 Research Design

**FIGURE 1:**
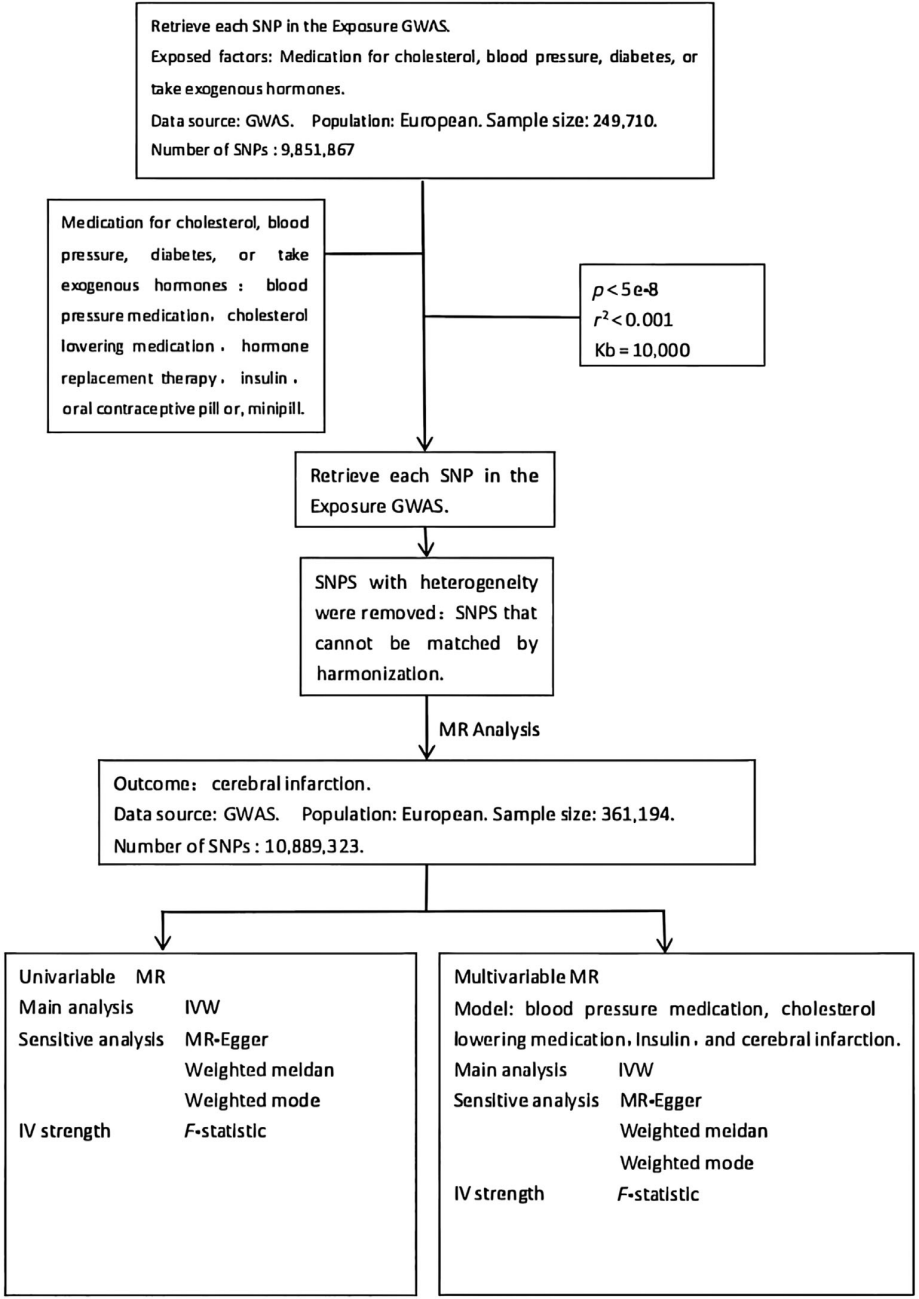
Study design and workflow.

A two‐sample MR analysis was employed to evaluate the causal impacts of cholesterol‐lowering medication, blood pressure medication, insulin, exogenous hormones, oral contraceptive pill, or minipill on cerebral infarction. The study design is illustrated in Figure 1, encompassing a total of five MR analyses. Initially, univariate MR was utilized to investigate the causal effects of these five drugs on cerebral infarction. Subsequently, multivariate MR models were developed to examine the direct or independent effects of using these drugs on the outcome.

### 2.2 Data Source

Our study utilized publicly available genome‐wide association study (GWAS) data that had been previously approved by informed consent and ethical review. We retrieved cerebral infarction patient data from the Neale lab from an online GWAS database. Information regarding cholesterol‐lowering medication, blood pressure medication, insulin use, exogenous hormone intake, oral contraceptive pill, or minipill was sourced from the MRC‐IEU. Further specifics of the GWAS data can be found in Table S1.

### 2.3 Selection of Tool Variables

MR in our study is grounded on three core assumptions: the correlation assumption, which posits that the IV, genetic variation (SNP), is closely associated with the exposure factors of interest (*p* < 0.05); the independence assumption, which asserts that the relationship between SNPs and confounding factors is independent of each other; and the exclusivity assumption, which states that the SNP does not have a causal relationship with the outcome but rather through the exposure effect (Davies, Holmes, and Davey Smith 2018). In the univariate MR analysis, SNPs with high significance levels (*p* < 5 × 10^−8^) were included, and horizontal pleiotropy between exposure and outcome was assessed. Confounding factors were addressed using the online tool LDtrai (https://ldlink.nih.gov/?tab=ldtrait) (Lin, Brown, and Machiela 2020), ensuring the validity of the independence assumption based on linkage disequilibrium (LD), with LD clustering *r* ^2^ < 0.001 and Kb = 10,000. Sensitivity analysis was conducted for each trait if the IVW result indicated *p* < 0.05. For multivariate MR analysis, a model was constructed incorporating the use of cholesterol‐lowering medication, blood pressure medication, insulin, and cerebral infarction. These traits were combined to mitigate the effects of confounding factors. Additionally, to avoid weak IVs, the *F*‐statistics were utilized as an assessment tool for IV strength, calculated using the formula *F* = data2/se2. When *F* > 10, it indicates a stronger correlation between exposure and IV, leading to a more accurate causal inference between exposure and outcomes (Burgess, Thompson, and CRP CHD Genetics Collaboration 2011).

### 2.4 MR Analysis

Univariate MR analyses were conducted, with a focus on the results of the inverse variance weighted (IVW) method, along with sensitivity analyses. Additional methods such as MR‐Egger (Burgess and Thompson 2017), weighted median (Bowden et al. 2016), weighted mode (Bowden et al. 2017), and MR‐PRESSO (Nikolakopoulou, Mavridis, and Salanti 2014) were employed to assess the heterogeneity and horizontal pleiotropy of SNPs to ensure the robustness of the analysis outcomes. Furthermore, a multivariate MR analysis was carried out on cholesterol‐lowering medication, blood pressure medication, and insulin with cerebral infarction, with IVW remaining the primary method of evaluation. In instances of positive findings, multivariate MR‐Egger (intercept), MR‐PRESSO, and weighted pattern tests were utilized to examine the heterogeneity and horizontal pleiotropy of the multivariate results. Heterogeneity in this context refers to the potential variations in the causal inferences of different SNPs on exposure and outcomes. In multivariate MR analysis, heterogeneity serves as a tool to assess the differences in the effects of SNPs.

## 3 Result

The study results utilize European population data, detailed information, and the relevance of SNPs as presented in Tables S2–S4. In the univariate MR analysis, the *F*‐statistic was computed using the formula *F* = beta2/se2, and all resulting *F* values exceeded 10. The dataset was deemed reliable and unaffected by weak IVs.

### 3.1 Univariate MR Analysis

**FIGURE 2:**
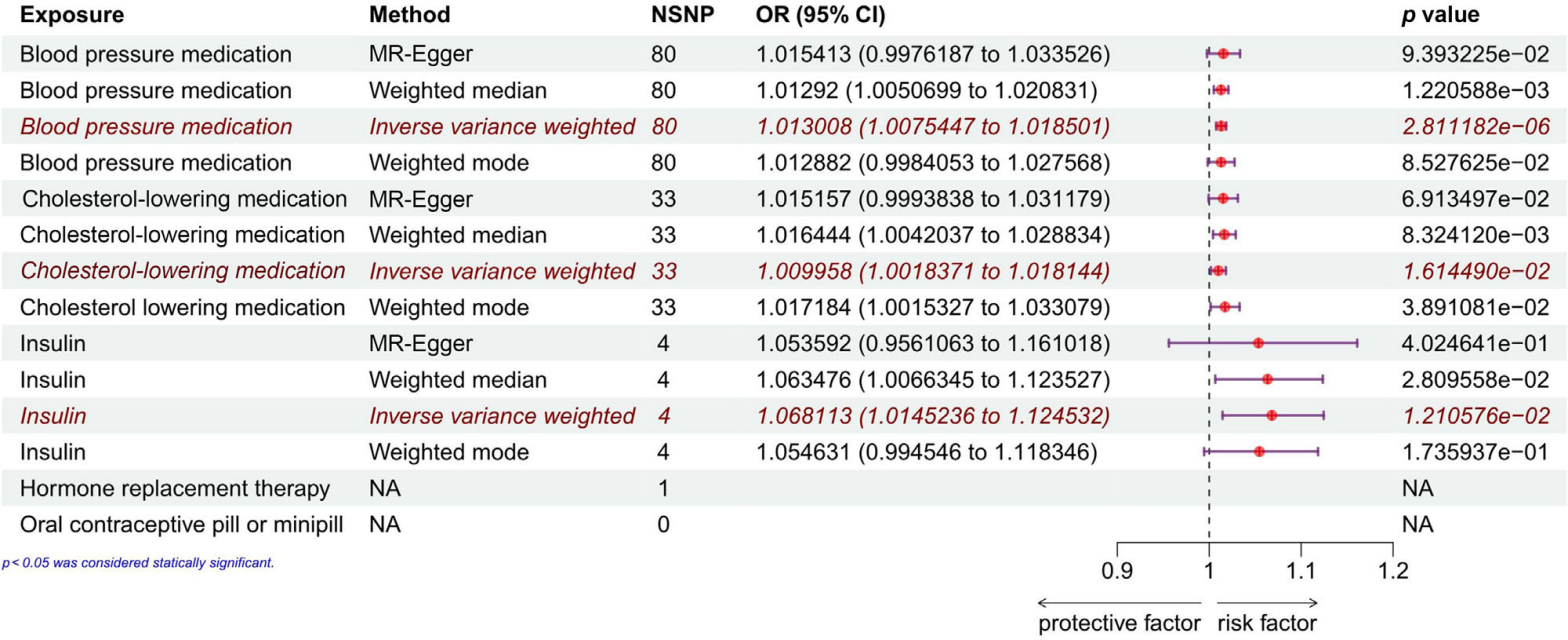
Univariate Mendelian randomization analysis of the causal relationship between cholesterol‐lowering medication, blood pressure medication, insulin, exogenous hormones (including hormone replacement therapy, oral contraceptive pill, or minipill), and cerebral infarction. IVW: inverse variance weighting method.

The study conducted an analysis on the effects of cholesterol‐lowering medication, blood pressure medication, insulin, exogenous hormones (including hormone replacement therapy, oral contraceptive pill, or minipill), and cerebral infarction using the inverse variance weighting (IVW) method as the primary approach. The results, depicted in Figure 1, were obtained, and the heterogeneity tests conducted through Cochran's *Q* test are presented in Table 1. The findings indicated no heterogeneity in the utilization of blood pressure medication and insulin (*p* > 0.05), leading to their analysis through a fixed‐effect model. Conversely, heterogeneity was observed in the SNPs of cholesterol‐lowering medication (*p* < 0.05), prompting the utilization of a random‐effect model for the IVW analysis. The results indicated that the heterogeneity did not impact the IVW outcomes, ensuring the reliability of the results. Furthermore, the sensitivity analysis did not reveal any horizontal pleiotropy in the SNPs within the exposure group, as detailed in Table 1. The leave‐one‐out analysis demonstrated that individual SNPs did not influence the causal inference, as illustrated in Figure S1. Effect estimates for individual SNPs in the study are provided in Figure S2. The univariate MR analysis (Figure 2) based on the IVW results suggested that prolonged and heavy use of blood pressure medication (OR: 1.013008 [1.0075447, 1.018501], *p* < 0.001), cholesterol‐lowering medication (OR: 1.009958 [1.0018371, 1.018144], *p* = 0.0161449), or insulin (OR: 1.068113 [1.0145236, 1.124532], *p* = 0.01210576) may elevate the prevalence of cerebral infarction. Moreover, the MR‐Egger's method and weighted mode supported a causal relationship between cholesterol‐lowering medication and cerebral infarction (*p* < 0.05). The weighted median method also endorsed the causal association between cholesterol‐lowering medication and cerebral infarction, with all *p* values being less than 0.05. Additionally, the study did not find any significant correlation between the use of exogenous hormones (including hormone replacement therapy, oral contraceptive pill, or minipill) and cerebral infarction. MR‐PRESSO was used to further exclude the abnormal SNPs (rs11591147, rs28383233, and rs73502335) when cholesterol‐lowering medication was used as the exposure, and no potential abnormal values were found for the remaining exposure factors. After our rigorous screening of SNPS and elimination of outliers, neither the MR‐Egger intercept test nor the MR‐PRESSO overall test found evidence of pleiotropy (Table 1).

**TABLE 1:** Results of heterogeneity and pleiotropy.

Exposures	SNPs	Heterogeneity				Pleiotropy			MR‐PRESSO	
		Q‐MR‐Egger	Q‐IVW	P‐MR‐Egger	P‐IVW	Intercept	SE	*p* value	Global test RSSobs	*p* value
Blood pressure medication	83	60.12103	60.19729	0.9336812	0.9429635	−2.29E‐05	8.29E‐05	0.7831631	67.26104	0.908
Cholesterol lowering medication	33	34.44237	35.07051	0.3064234	0.3245568	−6.64712E‐05	8.84036E‐05	0.4577789	39.26077	0.312
Insulin	4	1.178404	1.188208	0.5547698	0.7558339	2.11E‐05	2.13E‐04	0.930157	4.327112	0.66
Hormone replacement therapy	1	NA	NA	NA	NA	NA	NA	NA	NA	NA
Oral contraceptive pill or minipill	0	NA	NA	NA	NA	NA	NA	NA	NA	NA

### 3.2 Multivariate MR Analysis

Multivariable MR (MVMR) models were developed based on univariate MR analysis to investigate the causal association between cholesterol‐lowering medication, blood pressure medication, insulin, and the risk of cerebral infarction. The IVW method was used as the primary evaluation method to detect relevance. The results showed that under the influence of the independent effects of positive exposure. There was a significant positive causal relationship between blood pressure medication (OR: 1.009710 [1.0031017, 1.016361], *p* = 0.003921084), insulin (OR: 1.051087 [1.0016765, 1.102935], *p* = 0.042541027), and cerebral infarction. No significant association was found between cholesterol‐lowering medication and cerebral infarction (details are provided in Table 2). There was no significant causal relationship between cholesterol‐lowering medication and cerebral infarction (*p* > 0.05, Table 2). Horizontal pleiotropy was assessed using MR‐PRESSO, with a result of *p* > 0.05, indicating no horizontal pleiotropy.

**TABLE 2:** Results of multivariate Mendelian randomization analysis (SNP inclusion criteria *p* < 5E‐8).

Exposure	Outcome	NSNP	*p* value	OR	95% CI
Insulin (ukb‐b‐14070)	Cerebral infarction (ukb‐d‐I63)	270	0.042541027	1.051087	(1.0016765, 1.102935)
Cholesterol‐lowering medication (ukb‐b‐17805)		270	0.272638703	1.006300	(0.9950716, 1.017655)
Blood pressure medication (ukb‐b‐18009)		270	0.003921084	1.009710	(1.0031017, 1.016361)

In conclusion, our results showed a positive association between blood pressure medication and insulin with cerebral infarction. However, our current study does not support a causal relationship between cholesterol‐lowering medication, exogenous hormone administration (including hormone replacement therapy, oral contraceptive pills, or minipills), and cerebral infarction.

## 4 Discussion

To investigate the association between the misuse of common medications—such as Cholesterol‐lowering medication, blood pressure medications, insulin, and exogenous hormones—and cerebral infarction, we conducted a comprehensive MR analysis. Our univariate MR findings indicated a positive causal relationship between blood pressure medications, cholesterol‐lowering medication, insulin, and cerebral infarction. These results suggest that the misuse of blood pressure medications, cholesterol‐lowering medication, and insulin may serve as potential risk factors for cerebral infarction. Furthermore, due to the stringent limitations on SNPs, the current number of SNPs is insufficient to adequately support the investigation of the relationship between exogenous hormones—including hormone replacement therapy and oral contraceptives (such as minipills)—and cerebral infarction. To ensure rigorous and accurate results in MR, we strictly included SNPs with a significance threshold of *p* < 5 × 10^−8^ and did not expand the inclusion criteria for SNPs at this time. Further exploration will be conducted following future database updates. It is important to note that, considering the interactions among multiple drugs, we also employed MVMR. The results further confirmed a positive causal relationship between blood pressure medications, insulin, and cerebral infarction. However, the MVMR results did not indicate a significant association between cholesterol‐lowering medications and cerebral infarction. This study provides substantial evidence that the misuse of blood pressure medications and insulin may be a potential risk factor for cerebral infarction.

### 4.1 Accept the Hypothesis of the Result

In this study, we utilized single‐variable MR analysis combined with multivariate MR. In comparison to ordinary MR, our study further minimized the influence of pleiotropic and confounding factors on the study results. Pleiotropy refers to the phenomenon where one gene affects the development of multiple traits, which can significantly affect our causal inference in MR studies.

Cerebral infarction, mostly caused by atherosclerosis of intracranial and cervical large arteries, is prevalent in the elderly population and is a major public health challenge. Previous studies have suggested that cerebral infarction is closely related to intracranial blood pressure (Sun et al. 2024). Blood pressure medications play a crucial role in regulating and controlling blood pressure. However, many hypertensive patients tend to focus on the control of hypertension and underestimate the risk of hypotension, often increasing the dose of blood pressure medication on their own. This behavior can lead to persistent hypotension (Matsushime and Kuriyama 2021), and relevant studies have found that intracranial hypotension can also induce cerebral infarction (Kwon et al. 2022). A previous study on drugs and pharmacokinetics indicated that an overdose of blood pressure medication increases the risk of intracranial hypotension and is also likely to induce orthostatic hypotension in the elderly population (Dalakishvili et al. 2010). In patients with multiple comorbidities, hypotension resulting from improper combinations or excessive use of blood pressure medications is more prevalent (Alghalayini 2020). Although there are few documented cases of cerebral infarction directly caused by intracranial hypotension in clinical practice, it is undeniable that low intracranial blood pressure significantly elevates the risk of cerebral infarction. Magnetic resonance imaging reports have revealed that cerebral infarction is also common in patients experiencing severe hypotension (Tanaka et al. 2015). Additionally, a previous case report highlighted that cerebral infarction is one of the frequent complications associated with spontaneous intracranial hypotension (Redon et al. 2020). Another study suggested that sustained iatrogenic hypotension greatly increases the risk of cerebral infarction, with most cases being classified as watershed cerebral infarctions (Kurowski, Mullen, and Messé 2016). These studies collectively demonstrate the inextricable relationship between intracranial hypotension and cerebral infarction. Therefore, in conjunction with the results of our MR analysis and previous studies, we propose that an overdose of blood pressure medication heightens the risk of orthostatic hypotension, and the frequent occurrence of orthostatic hypotension may lead to cerebral infarction. A meta‐analysis involving 121,913 individuals indicated that orthostatic hypotension is associated with a 64% increased risk of stroke (Moreira 2020), further substantiating our hypothesis. Furthermore, excessive or improper combinations of blood pressure medications may also result in prolonged intracranial hypotension. Under the influence of hemodynamics, if intracranial hypotension is not corrected within a certain timeframe, it can lead to tissue hypoperfusion, and sustained brain tissue hypoperfusion may also induce cerebral infarction.

Insulin is secreted by islet beta cells and functions as a hypoglycemic hormone. However, a Chinese study of hospitalized patients with type 2 diabetes found that some type 2 diabetics using long‐acting insulin developed hypoglycemia, suggesting that improper insulin use may have side effects associated with hypoglycemia (Qiang et al. 2024). Clinical studies have found that intensive insulin therapy after acute ischemic stroke cannot only reduce blood glucose levels but can also lead to the aggravation of cerebral infarction in patients (Rosso et al. 2012). In addition, a study of insulin therapy with a glycemic target of 2–4 mmol (−1) found that insulin therapy at this target promoted cerebral infarction and increased mortality in patients (Meden et al. 2002). In this investigation, all participants receiving insulin treatment demonstrated significantly reduced blood sugar levels. Taken together, these findings suggest that hypoglycemia induced by inappropriate insulin dose may induce or aggravate cerebral infarction in patients, which further supports our MR results. A case report pointed out that improper insulin dosage can cause hypoglycemia and induce takotsubo syndrome, which leads to cerebral infarction (Koyama et al. 2020). This may be one of the potential mechanisms of insulin abuse leading to cerebral infarction. Based on previous studies, hypoglycemia can lead to neurological deficits, which may be associated with acute brain injury (Shirayama et al. 2004), cerebral vasospasm (Sakurai et al. 2023), or asymmetry in cerebral blood flow (CBF) (Sontineni, Lee, and Porter 2008). A study examining hypoglycemia and pontine infarction analyzed 10 regions of the brain in healthy men during insulin‐induced hypoglycemia. The findings revealed that the increase in CBF in the pons/brainstem was the lowest, whereas the increases in other regions were relatively high. Under this hypoglycemic blood flow pattern, patients with basilar artery stenosis were more susceptible to pontine infarction (Sontineni, Lee, and Porter 2008). The abnormal changes in CBF during hypoglycemia may represent another potential mechanism for cerebral infarction induced by insulin abuse. This association could explain cerebral infarction induced or exacerbated by excessive insulin use, consistent with the results of our comprehensive MR analysis. MR analysis outcomes provide a theoretical basis for these findings.

This study investigated the effects of cholesterol‐lowering medication, blood pressure medication, insulin, and exogenous hormones on cerebral infarction. By analyzing the available data, a significant positive causal relationship between the misuse of blood pressure medication, insulin, and cerebral infarction was determined.

## 5 Conclusion

The findings from MR analysis indicated that the misuse of blood pressure medication and insulin could be a potential risk factor in the onset and progression of cerebral infarction. The results of the analysis offer genetic support for a causal association between blood pressure medication, insulin, and cerebral infarction. In clinical practice, personalized approaches and appropriate utilization of blood pressure medication and insulin could serve as effective strategies for the prevention and management of cerebral infarction.

## 6 Limitations

Our research primarily focused on European populations. To validate our results, it is essential to replicate MR analyses in diverse racial and regional groups due to the influence of race and region. Although some studies have indicated that cholesterol‐lowering medications can decrease the likelihood of cerebral infarction (Lee et al. 2020), our multivariate MR analysis based on 2018 data did not reveal its independent impact. Further investigation may be necessary once the database is refreshed.

## Author Contributions

**Jing Liu**: conceptualization, writing–original draft, writing–review and editing, investigation, validation, methodology, software, data curation, resources, visualization. **Dongsheng Lv**: methodology, software, validation. **Shiduo Li**: validation, methodology, visualization. **Zhansen An**: validation, software, visualization. **Zefeng He**: software, supervision, data curation, validation. **Yingzi Liu**: conceptualization, methodology, validation, software, formal analysis, writing–review and editing.

## Conflicts of Interest

The authors declare no conflicts of interest.

### Peer Review

The peer review history for this article is available at https://publons.com/publon/10.1002/brb3.70186.

## Supporting information



Supplementary Materials.

Supplementary Materials.

Supplementary Materials.

Supplementary Materials.

Supplementary Materials.

Supplementary Materials.

## Data Availability

Publicly available datasets were analyzed in this study. These data can be found here at https://gwas.mrcieu.ac.uk/datasets/.
