# Supplementary figures and images for: Causal Relationship Between the Abuse of Cholesterol‐Lowering Medication, Blood Pressure Medication, Insulin, and Exogenous Hormones and Cerebral Infarction
Source: Brain Behav. 2024 Dec 22;14(12):e70186. doi: 10.1002/brb3.70186 (PMC11663836; doi:10.1002/brb3.70186)

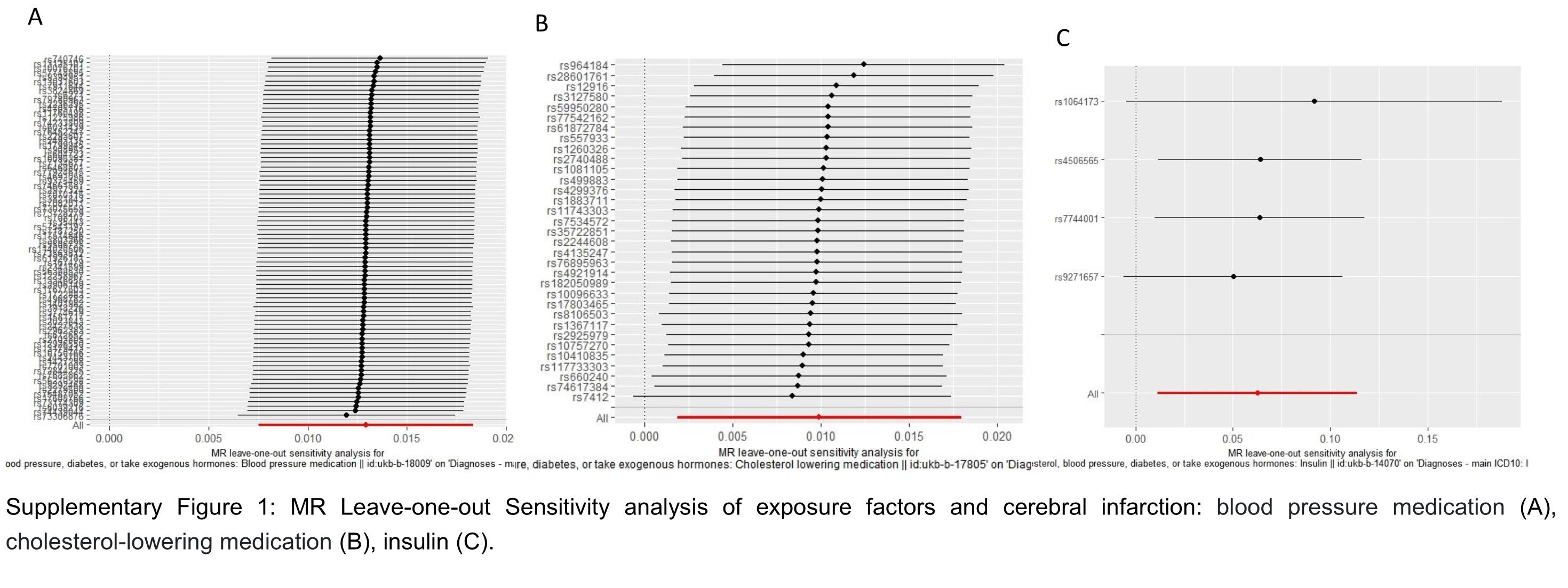

Supplement: Supplementary file 1 — Supplementary Materials. [file BRB3-14-e70186-s005.Jpeg]

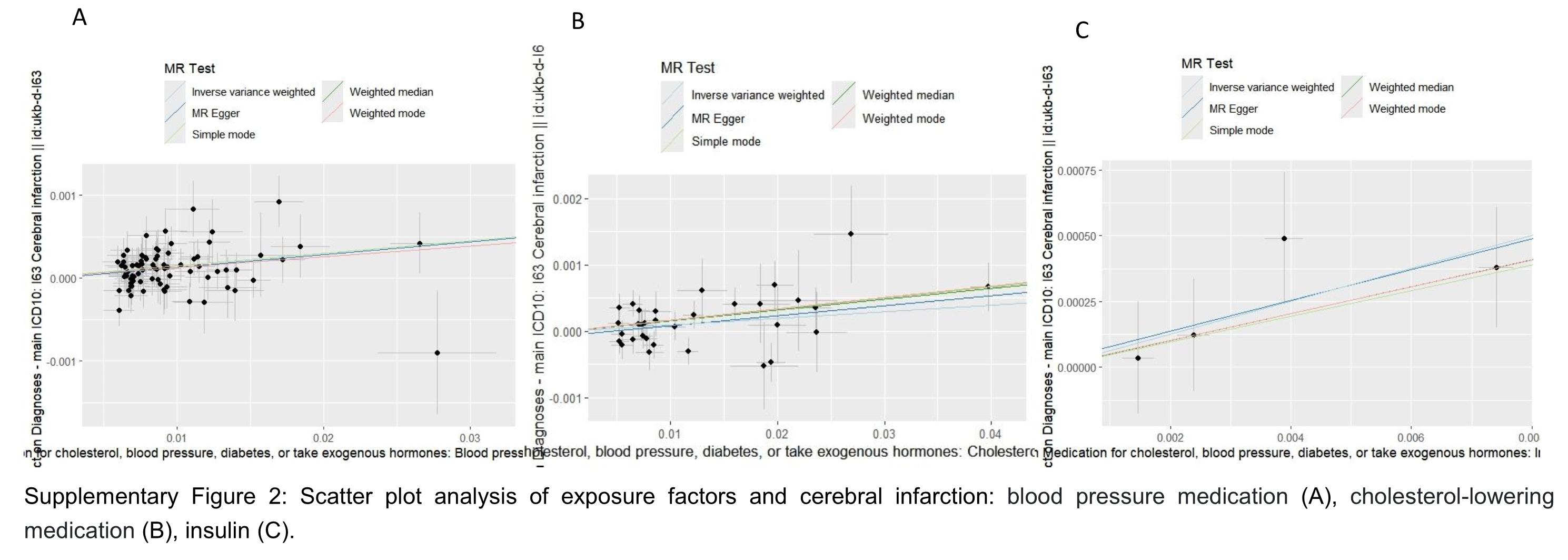

Supplement: Supplementary file 2 — Supplementary Materials. [file BRB3-14-e70186-s006.Jpeg]
